# Bennett’s Fracture Management: A Systematic Review of Literature

**DOI:** 10.7759/cureus.31340

**Published:** 2022-11-10

**Authors:** Poornanand Goru, Syed Haque, Gopalkrishna G Verma, Abubakar Mustafa, Ananthan Ebinesan

**Affiliations:** 1 Trauma and Orthopaedics, Manchester Royal Infirmary Hospital National Health Service (NHS) Trust, Manchester, GBR; 2 Trauma and Orthopaedics, Manchester University National Health Service (NHS) Foundation Trust, Manchester, GBR

**Keywords:** systematic review, management, thumb metacarpal, thumb cmc, bennett’s fracture

## Abstract

Bennett’s fracture is a relatively common fracture of the base of the thumb with no consensus on optimum management. Determining the optimal treatment method for Bennett’s fractures remain a challenge and has been the subject of much debate.

This systematic review aims to investigate present and past literature and determine the optimum treatment intervention for Bennett’s fracture-dislocation. The primary outcome measure is post-traumatic arthritis, and the secondary outcome measures are reoperation, pain, infection, and nonunion.

As per Preferred Reporting Items for Systematic Reviews and Meta-Analyses (PRISMA) guidelines, a systematic review of the literature was performed to evaluate patient demographics, clinical profile, management, and treatment outcomes. Two authors independently performed electronic searches of the Embase, Medical Literature Analysis and Retrieval System Online (MEDLINE), and Cochrane databases. Studies conducted between 1963 and 2021 with articles reporting Bennett’s fracture management were included. The study was registered with PROSPERO (CRD42021295464).

In the initial screening, 58 articles were identified, of which 13 articles met the criteria and were included in the final review, evaluating 558 patients. Out of these, 439 were managed by various surgical procedures, and 119 were treated by manipulation and plaster of Paris (POP) immobilization. Of the 13 studies considered, eight have a clear mention of post-traumatic osteoarthritis, with a total of 50 (9%) patients. Secondary outcomes included pain in 76 (13%) patients, infection in four patients, reoperation in 11 (2%) patients, and no nonunion.

This review was conducted with the help of retrospective studies as there is no randomized controlled trial on the management of Bennett’s fracture. Our primary outcome measure of post-traumatic arthritis in patients being treated by operative and conservative methods was mentioned in these studies. However, due to the sample size being small and the heterogenicity of these studies, the strength of these findings is low. Due to these shortfalls, this review study cannot recommend any single (or) particular treatment for all patients with Bennett’s fracture.

## Introduction and background

Bennett’s fracture is a fracture subluxation of the thumb carpometacarpal joint (CMCJ), with a sizable palmar anterior marginal fracture fragment. The fracture is named after Edward Hallaran Bennett [1], a professor of surgery at Trinity College, Dublin. He described it as a fracture that “passed obliquely across the base of the bone, detaching the greater part of the articular surface.” He further mentioned that rather than a fracture, this looked like a dorsal subluxation of the first metacarpal [1].

Fractures of the metacarpal bones account for 10% of all fractures. This is nearly 40% of all hand fractures [2,3]. The lifetime incidence of metacarpal fracture is 2.5%. Fractures involving the articular surface of the carpometacarpal joint are the most common of all thumb fractures [1,4-6]. The male-to-female ratio for this type of fracture is 10:1 [7]. This fracture is more common in the younger age group, with nearly half of them happening in patients less than 30 years of age. Thumb fracture accounts for 25% of all metacarpal fractures and is the second most common metacarpal injury after fracture of the metacarpal neck of the little finger [8,9]. Of these thumb metacarpal fractures, 80% are at the base. Thumb accounts for 40% of hand function, and given this, these injuries and their management are of significant interest.

The thumb carpometacarpal joint (CMCJ) is a saddle-shaped joint. Due to its anatomy, it allows greater movement to the thumb. However, unlike other CMCJ of the hand, the only bony attachment of the thumb CMCJ is the metacarpal of the thumb to the trapezium. This joint is completely isolated from other CMCJ of the hand, and this renders it the most unstable of all the CMCJ.

The lack of bony attachment is supplemented with the ligaments that provide stability to the thumb CMCJ. Among the many ligaments of the thumb, CMCJ’s two important ligaments are the dorsoradial ligament, which prevents dorsomedial subluxation, and the beak ligament, which prevents dorsal subluxation. The mechanism of injury causing these fractures is axial loading on a flexed thumb. This causes metacarpal base fracture with the volar fragments still articulating with the joint, but the fracture distal to this causes subluxation of the thumb CMCJ due to the pull of the abductor pollicis longus and the slope of the trapezium [6].

Traditionally, the management of these fractures has been controversial and, in many cases, has resulted in suboptimum results. There is no consensus among orthopedic surgeons regarding the treatment of this fracture. Bennett himself was dissatisfied with the outcome of these fractures, which left his patients failing to grasp or lift things with their hands two years following the injury [1].

Like all other intra-articular injuries, there was a push in the 20th century for anatomical reduction of joint surfaces for the management of these fractures. This came from the background of studies from the past that showed that nonoperative management of Bennett fracture resulted in post-traumatic arthritis of the thumb [8]. Many ways were used for the anatomical reduction of the thumb CMCJ, which included plaster of Paris (POP) cast immobilization and traction of the thumb [10,11], K-wire fixation, and open reduction and plate fixation of the fracture [12]. However, many studies showed that the outcome can be surprisingly very good with a functional hand even with the imperfect reduction of the joint [6,13].

Currently, there is no consensus on the optimum management of Bennett’s fracture. However, one thing is clear: the joint needs to be reduced for good functional outcomes following this fracture-dislocation [13]. This systematic review aims to investigate present and past literature to determine the optimum operative management of Bennett’s fracture-dislocation. This study looked into adult patients who had isolated closed Bennett’s fractures following trauma, which was managed with any of the following interventions: manipulation under anesthesia (MUA) and POP application or invasive surgical interventions such as K-wire fixation or open reduction and internal fixation (ORIF). The primary outcome measure is the development of post-traumatic arthritis. The secondary outcome measures are reoperation, pain, infection, time to union, and nonunion.

## Review

Methodology

Search Strategy

The databases Cochrane Library (Cochrane Systematic Reviews and Cochrane Bone, Joint, and Muscle Trauma Group), PubMed, Embase, Cumulative Index to Nursing and Allied Health Literature (CINAHL), ScienceDirect, Google Scholar, and ISI Web of Knowledge were used to conduct a literature search. Articles published between 1963 and 2021 were considered. There were no language restrictions.

Search Terminologies

Medical Subject Headings (MeSH) terms from the National Library of Medicine (NLM) were chosen and used alongside text words. The MeSH phrases “Bennett’s fracture,” “Thumb,” and “Thumb carpometacarpal joint” were utilized to give a systematic approach to access information when more than one author used different terms for the same idea. “Bennett’s fracture” OR “Bennett’s fracture dislocation” OR “Bennett’s fracture management” AND “Thumb carpometacarpal joint” AND “Bennett’s fracture outcomes” were among the terms used.

Inclusion Criteria

**Figure 1:**
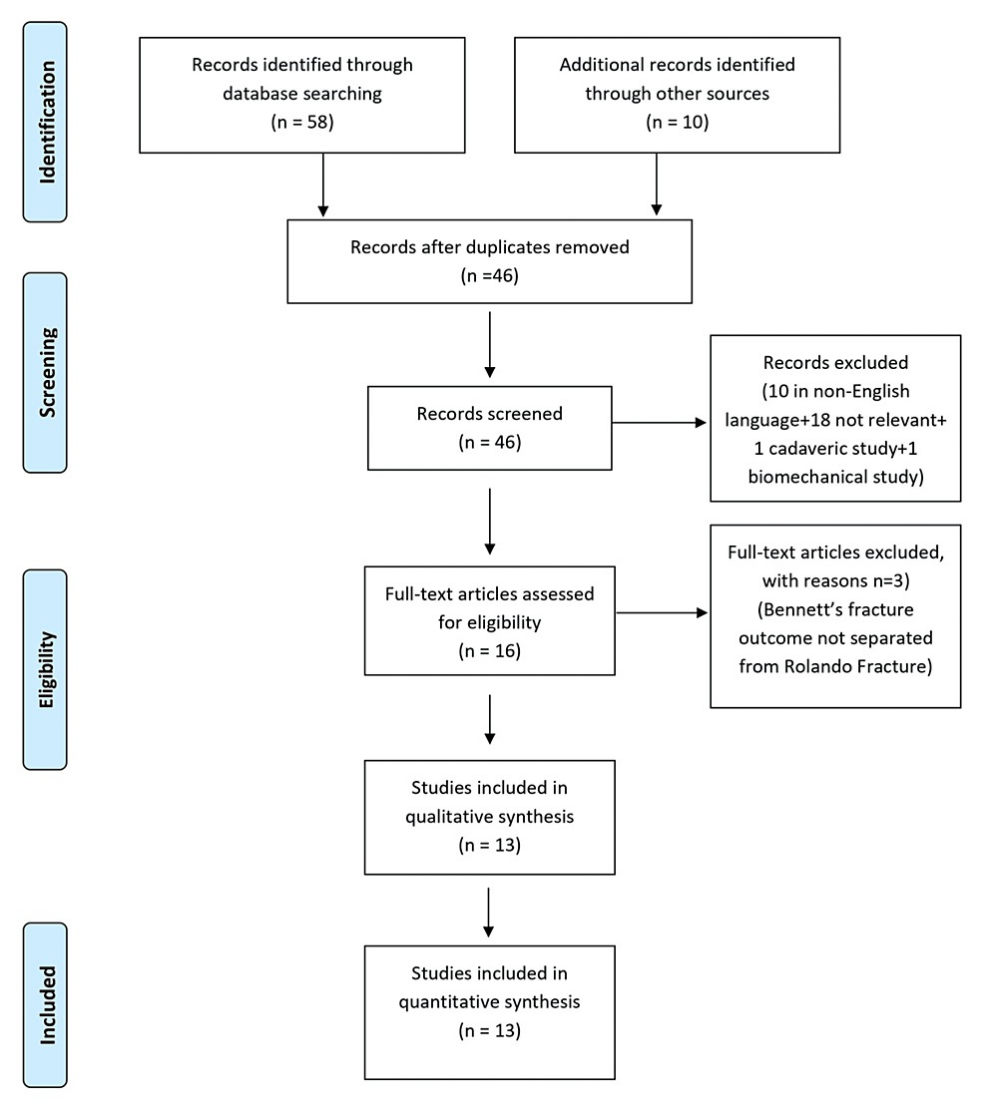
PRISMA Flow Diagram PRISMA: Preferred Reporting Items for Systematic Reviews and Meta-Analyses

Articles on the result of Bennett’s fracture-dislocation were included. Following the application of exclusion criteria, all pertinent abstracts were assessed, and the whole text of the selected publications was obtained. Figure 1 depicts the Preferred Reporting Items for Systematic Reviews and Meta-Analyses (PRISMA) flow diagram of the approach used. Two writers (SH and PG) did the literature search, and there were no conflicts about which articles were included.

Exclusion Criteria

Articles that reported on the outcomes of Bennett’s fracture care in children, polytrauma, cadaver studies, and animal studies, as well as research that did not provide a specific statement of our primary outcome, were omitted. Articles written in languages other than English were not considered. Duplicate articles were not accepted.

Quality Appraisal

Two authors objectively appraised the quality of the selected studies and evaluated their results using the Population, Intervention, Comparison, and Outcome (PICO) method, Critical Appraisal Skills Programme (CASP) tools, and Methodological Index for Non-randomized Studies (MINORS). Because no randomized controlled trials were available, the papers that were considered were retrospective and had a significant risk of bias. The risk of bias tool was used separately by authors SH and PG, and disagreements were resolved through discussion with the third review author (GV).

Results

In the first phase, 68 articles were identified from the searched databases, and in the second phase, 13 articles that met the inclusion criteria were chosen for final inclusion. One prospective study and 12 retrospective studies were included. The studies were conducted between 1963 and 2021. Thirteen studies comprised a total of 558 patients; 439 of these patients were managed by various surgical procedures, and 119 were treated with manipulation and plaster of Paris (POP) immobilization. In all studies, the patients’ mean age was 35.2 years, with the youngest patient being 16 years old and the oldest being 75 years old. The male-to-female ratio is 9:1. Road traffic accidents, sports injuries, falls, and physical aggression are the most common causes of injury. Only eight of the 13 studies mentioned follow-up, with durations ranging from one to 26 years. Patient satisfaction was reported to be 92% in nine trials. Seven studies reported return to work within six weeks of having the cast removed.

Primary Outcome: Post-traumatic Arthritis

The primary outcome measure of this systematic review was radiological evidence of arthritis at the final review. Eight out of 13 studies have a clear mention of post-traumatic arthritis, totaling 50 (9%) patients. In the management of Bennett’s fracture, there was no significant difference in the incidence of post-traumatic osteoarthritis between conservative and surgical interventions.

Secondary Outcomes

In this review, patients with residual pain were described in 76 (13.6%) studies. Out of all surgical intervention patients, seven developed a superficial infection, of which six responded to oral antibiotics and one required intravenous antibiotics and wound debridement. Eleven (2%) patients underwent reoperation, in which six patients failed manipulation and cast, and another five patients were in the operative group. Nonunion was not reported in the included studies.

Discussion

The objective of this systematic review was to compare operative and nonoperative interventions and the outcome of the management of Bennett’s fracture. There is no randomized controlled trial done comparing operative versus nonoperative management of Bennett’s fracture. The majority were retrospective case series, and very few compared different treatment modalities. Patients were mostly followed up in the clinic, and in a few cases, outcome measures were collected retrospectively from case notes. The small number of eligible studies and variation in outcome measures between studies meant that the pooling of data was only feasible for a minority of secondary outcome measures. There was no validated scoring system used to quantify either the primary or secondary objectives.

Our primary outcome measure was evidence of post-traumatic osteoarthritis on follow-up examination in patients undergoing treatment for Bennett’s fracture. In the studies selected for this systematic review, none of the authors had used any validated clinical scoring system to diagnose osteoarthritis. The only method used for diagnosing post-traumatic osteoarthritis in these studies was the radiological assessment of the trapezio-metacarpal joint of the thumb.

Post-therapy pain was a common factor in all operative interventions, and the incidence was similar in operative and nonoperative interventions. Griffiths and Cannon et al. report a reduction in abduction in a significant proportion of patients [13-16]. The majority of studies, except for Cannon et al. [16], reported good opposition. Fracture healing was in varus alignment in the study of Griffiths [13] and Cannon et al. [16] in a number of patients; however, these did not correlate with pain or eventual post-traumatic arthritis. These data could not be combined since no standard deviation was provided.

No study presented data on various patient groups accompanying Bennett’s fracture, e.g., sports people, desk workers, or manual laborers. Hence, these groups could not be analyzed separately.

Robinson [14] has pointed out that observer variation can be substantial and should be taken into account when radiological diagnostic methods are considered; in many cases, the dissimilarity between observers outweighs the difference between techniques. In these studies, there was no mention of whether the assessors who interpreted the X-rays had formal training or not.

Post-traumatic arthritis was not found in two studies in which operative intervention was done. However, in the study of Brüske et al. [15], where percutaneous K-wire was used, there was a significantly high percentage of patients who developed osteoarthritis. There was no meaningful dissimilarity in the occurrence of post-traumatic osteoarthritis in patients managed operatively or nonoperatively.

Pain was a constant finding in 11 out of 13 studies being considered in this systematic review. There is wide variation in reporting pain between studies. Nine out of 13 studies have mentioned the range of movement at the trapezio-metacarpal joint. After pooling the data, it was found that the movement that was significantly affected was the abduction of the thumb. Most of the patients achieved good opponency movement despite the shortening of the thumb in a few cases. More importantly, few studies found no correlation between the severity of post-traumatic arthritis and the range of movement at the trapezio-metacarpal joint.

Cannon et al. [16] and Bennett [1] mentioned fracture alignment at the final checkup. In both of these studies, fracture was managed by closed reduction, and position was maintained by plaster of Paris cast. The predominant malunion was a varus deformity.

Reoperation is expensive both in monetary and personal terms. All the studies except, Zhang et al. [17], have mentioned the number of patients undergoing reoperation. After excluding the study of Zhang et al., pooling of data was done, which showed that of 439 patients who underwent surgical intervention, five had to undergo reoperation. On the other hand, out of 109 patients who underwent manipulation under anesthesia and POP immobilization, six had to have their operation revised.

Time to union and return to normal activity had been mentioned in a few studies. Most of the fractures healed in 4-6 weeks. In the study of Pollen [6], all patients managed by MUA and POP cast returned to work eight weeks following the removal of the cast with the vast majority returning to work within four weeks.

The study of Spêngberg and Thorén [12] involved 34 patients treated with traction and K-wire fixation, which resulted in residual pain in two patients without any further complications related to surgery and outcome. The study of Griffiths [13] involving 21 patients treated with manipulation and cast application reported seven patients with residual pain and one patient with arthritis.

The study of Pollen [6] involved 31 patients who have been managed considerately with manipulation and cast without any significant complications. The study by Cannon et al. [16] included 21 patients with a plaster following manipulation and presented two post-traumatic arthritis and two patients with residual pain after a 10-year follow-up.

In a retrospective case series, Livesley [18] reported significant post-traumatic arthritis in 15 patients and residual pain in 13 patients out of 31 patients. Only this study has a considerably high number of complications than the rest of the studies. Kjaer-Petersen et al. [19], in their retrospective case-control study with conservative and operative treatments, out of 41 patients, 10 patients had the last follow-up; 10 had post-traumatic arthritis as well as residual pain. Two superficial infections were treated with oral antibiotics.

Timmenga et al. [20], in their retrospective case-control study, operated on 18 patients, of which six patients develop arthritis and another six patients had residual pain. The study of Pavić and Malović [21] included 89 patients with operative intervention; 10 patients with no post-traumatic arthritis developed residual pain.

In the prospective study of Mahmoud et al. [22] that included 10 patients with surgical intervention, pain score improved along with function. Middleton et al. [23] had the largest number of patients (143 patients). The patients were treated with manipulation and K-wire fixation. The study did not mention any post-traumatic arthritis; however, there are six infections, out of which one needed IV antibiotics and debridement. They reported excellent functional outcomes and a high level of satisfaction rate of 94%.

In the retrospective study of Pomares et al. [24], out of 21 patients, 11 patients were treated with arthroscopically assisted percutaneous screw fixation, and 10 patients underwent open reduction and screw fixation. Residual pain was present in six patients out of 10 patients in the open reduction group.

Limitations

All our included studies are mainly retrospective case-control studies, except one, which is prospective. The limitation of our study is that the number of patients included in the selected studies was small. Also, there are no validated outcome scores.

## Conclusions

As this review includes mainly retrospective studies, our primary outcome of post-traumatic arthritis is similar in the operative versus nonoperative group. Our secondary outcome measure of residual pain was significantly higher in patients managed with conservative options. However, due to the smaller sample size and heterogenicity of these studies, the strength of these findings is low. A multicenter randomized controlled trial of different interventions is warranted.
